# Supplementary material for: The brittle star genome illuminates the genetic basis of animal appendage regeneration
Source: Nat Ecol Evol. 2024 Jul 19;8(8):1505–21. doi: 10.1038/s41559-024-02456-y (PMC11310086; doi:10.1038/s41559-024-02456-y)
Supplement: Supplementary file 1 — Supplementary Notes 1–3 and Tables 1–14. [file 41559_2024_2456_MOESM1_ESM.pdf]

nature ecology & evolution

Supplementary information https://doi.org/10.1038/s41559-024-02456-y

The brittle star genome illuminates the genetic basis of animal appendage regeneration

In the format provided by the authors and unedited

Supplementary Notes

Note S1: Comparisons with previous efforts to sequence genomes from the brittle star group

Note S2: Tandem duplications of key genes likely contribute to brittle star larval skeleton and bioluminescence

Note S3: Genome annotation pipeline, gene list curation and Hox genes identification

Supplementary Tables

Table S1: Accessions for A. filiformis RNA-seq datasets.

Table S2: *A. filiformis* gene models properties.

Table S3: *A. filiformis* genes and signalling pathways.

Table S4: *A. filiformis* immune genes categories.

Table S5: List of DNA-binding PFAM domains.

Table S6: Enriched gene ontology terms in brittle star expanded and contracted gene families.

Table S7: Gene expansion and contraction and expression in regeneration.

Table S8: Co-expression clusters and expression of known brittle star regeneration genes.

Table S9: Co-expression clusters and expression of novel brittle star regeneration genes.

Table S10: Enriched gene ontology terms in brittle star arm regeneration co-expression clusters.

Table S11: Axolotl limb regeneration co-expression clusters.

Table S12: Enriched gene ontology terms in axolotl limb regeneration co-expression clusters.

Table S13: Genes with conserved expression profiles during appendage regeneration in brittle star, axolotl and Parhyale.

Table S14: Enriched gene ontology terms in genes with conserved expression profiles during appendage regeneration.

Supplementary Datasets

The following datasets have been deposited in Zenodo [1] :

Dataset S1: Gene and repeat annotation (including .fasta genome file, .bed, .gtf and .fasta files for *A. filiformis* genes and repeat annotations for the four genomes presented in Figure 1D). [Dataset_s1]

Dataset S2: Annotation of selected echinoderm genomes with respect to the predicted Eleutherozoa ancestral Linkage Groups (.bed files with genes annotated with their ancestral chromosome of origin and orthologous genes files). [Dataset_s2]

Dataset S3: Hox and ParaHox genes in A. filiformis (including Hox protein sequences and molecular phylogeny, and ParaHox sequences). [Dataset_s3]

Dataset S4: Echinoderm gene families, results of the expansion/contraction tests and alignments and trees for the luciferase and *pmar1/phb* gene families. [Dataset_s4]

Dataset S5: Raw counts and normalised gene expression tables for *A. filiformis* development, regeneration time course and explant experiments, the *A. mexicanum* limb regeneration time series and predicted homologs between *A. filiformis, A. mexicanum and P. hawaiensis.* [Dataset_s5]

Supplementary Notes

Note S1: Comparisons with previous efforts to sequence genomes from the brittle star class Ophiuroidea.

To date, and excluding our newly-generated high-quality *A. filiformis* genome, only three ophiuroid species have a draft genome assembly in the NCBI genome database: *Ophioderma brevispina*, *Ophionereis fasciata* and *Ophiothrix spiculata*. Only two of these genomes have been previously published: *Ophioderma brevispina* [2] and *Ophionereis fasciata* [3]. Critically, none of these sub-chromosomal assemblies have been found sufficiently robust for inclusion in the authoritative Database Echinobase [4]. Producing high-quality genomic resources for the brittle star class has thus represented a significant challenge, probably due to their relatively high genome size compared to other echinoderms, elevated repeat content and high level of heterozygosity. Based on long-read nanopore reads and proximity ligation data, and refinement with illumina short reads, our *A. filiformis* genome greatly improves upon the previously published ophiuroid draft genomes. The *A. filiformis* assembly has markedly higher completeness (96.1% vs 30% assembly complete BUSCO) and contiguity (68.8 Mb vs 48.5 kb N50) as well as a more realistic number of predicted genes (30,267 vs 146,703) than *Ophioderma brevispina* [2]. Similarly, the provided assembly statistics from the low-coverage *Ophionereis fasciata* genome [3] (N50: 72.8 kb; genes number: 102,838) showcase the significant improvements of our *A. filiformis* genome over the state-of-the-art.

Note S2: Tandem duplications of key genes likely contribute to brittle star larval skeleton and bioluminescence

Tandem gene duplications and subsequent asymmetric divergence are widespread in the evolution of animal genomes and have been linked to the evolution of species-specific traits. In echinoderms, two specific gene families have previously come into focus as relevant examples of lineage-specific evolution through tandem duplications: *phb*/*pmar1* (larval skeleton) and luciferases (bioluminescence) [5,6]. Pmar1 is the most upstream zygotic factor of the regulatory network controlling the specification of skeletogenic cells in sea urchins [7]. Among Eleutherozoa, only sea urchins and brittle stars develop an elaborated larval skeleton. In sea urchins, the *pmar1* gene originated through repeated lineage-specific duplications of an ancient phb paired-class homeobox gene. Duplications of the *pmar1* gene have been pinpointed as important drivers for the establishment of this sea urchin-specific regulatory programme, which culminates in the formation of the larval skeleton. In the brittle star *A. filiformis*, we identify a similar expansion of *phb* paralogs (totaling 13 phb genes). Phylogenetic analysis confirms that these *phb* paralogs are distant homologs of the sea urchins *pmar1*, but indicates that they are distinct from the previously described brittle star *pplx* gene [8] (**Extended Data Fig. 5A**). Moreover, expression of the *A. filiformis phb* genes occur largely during early development (**Extended Data Fig. 5E**), as described for the *pmar1* sea urchin gene. This suggests that the convergent evolution of brittle stars and sea urchins larval skeleton may have been driven by independent duplications of *phb* genes.

Brittle stars stand out by their ability to emit light. In *A. filiformis*, bioluminescence is mediated by a specific type of luciferase which is homologous to the well-characterised luciferase of the soft coral *Renilla reniformis* [5]. Within the *A. filiformis* genome, we identified nine luciferase-like gene copies: seven are organised in two clusters of tandem duplicates, two are isolated copies. This corroborates and extends the previously-inferred repertoire [5]. We find that luciferase-like genes have duplicated not only in the brittle star but also in all echinoderm lineages with the exception of sea stars (**Extended Data Fig. 5**). These results confirm previous propositions that echinoderms harbour multiple copies of luciferase-like genes, which likely encode diverse functions across bioluminescent and non-bioluminescent species, and will benefit from future in-depth functional characterisation.

Note S3: Genome annotation pipeline, gene lists curation and Hox genes identification

Genome annotation

We first assembled a consensus transcriptome from all RNA-seq samples with mikado [9], combining an alignment-free transcriptome assembled with Trinity [10] and mapped to the genome with gmap [11] with an alignment-based transcriptome mapped to the genome with STAR [12], assembled with Stringtie [13] and merged with taco [14]. Second, we selected best-scoring mikado transcripts (i.e. transcripts with identified start and stop codons by TransDecoder, at least 2 exons, over 50% of the predicted coding sequence covered by a swissprot [15] blast hit and no overlap of the coding sequence with an annotated repeat) to train a gene prediction model with AUGUSTUS [16]. Third, we obtained similarity-based gene predictions with Metaeuk [17], based on proteomes from a total of 27 metazoa, including 8 echinoderms and 2 hemichordates. Fourth, we performed *ab initio* gene prediction with AUGUSTUS, using the previously trained model and providing as hints the predicted exons by mikado and Metaeuk, and curated splice junctions defined by portcullis [18] on the STAR transcriptome. Fifth, we filtered out all predicted gene models with over 40% of exons overlapping annotated repeats and then ran PASA [19] on retained genes to finalise models and annotate UTRs. Finally, we further filtered out 3,465 poorly supported gene models (no PFAM domain, no swissprot blast hit and maximal expression < 2 TPM), to retain 30,267 annotated gene models in the final annotation. The annotation had a final score of quality and completeness of the annotation is demonstrated by a score of 92.7% complete BUSCO (C:92.7 [S:86.2%, D:6.5%], F:5.0%, M:2.3%, n:954) 137 and a total of 4,974 unique PFAM domains 138, with 76% of genes (23,047) containing a PFAM domain. Genes were named by BLAST search against the swissprot database. The names of genes specifically investigated in this study (*hox*, *phb*, *luciferase*) were further manually curated to reflect their evolutionary history.

Gene lists curation

We generated curated gene lists for *A. filiformis* (Table S2). For immune genes, we used a combination of PFAM domain annotation and lists of previously curated immune genes in the sea urchin *Strongylocentrotus purpuratus* [20]. Specifically, we first selected A. filiformis genes based on their PFAM domains (e.g. TIR, IL17, Mif) and completed this list by searching for homologs (using the set of broccoli gene families) with the immune genes of the sea urchin Strongylocentrotus purpuratus. PFAM domains were annotated on protein sequences using the pfam_scan.pl tool run against the PFAM-A HMMs database, with default parameters. We identified kinase genes through homologies with curated kinase genes from the sea urchin genome [20]. We generated a list of TFs based on the presence of DNA-binding PFAM domains (Table S2). For the stemness genes, we identified putative homologues of the 180 "stemness-like" genes established by ref [21], that is, genes that are shared between three stem cell populations: poriferan (*Ephydatia fluviatilis*) archeocytes, Hydra vulgaris interstitial stem cells, and planarian (*Schmidtea mediterranea*) neoblasts. Specifically, we used the human cognates of all those genes as queries for BLAST searches [22] against *A. filiformis* genes. For genes involved in neuronal function, we first compiled a list of neurogenic and glial markers, TFs and cell signalling genes involved in embryonic, homeostatic, and regenerative neurogenesis in vertebrates (rodents, humans, and Xenopus) and invertebrates (*Caenorhabditis elegans* and

Drosophila melanogaster). We identified putative homologues of "neuronal" genes in *A. filiformis* using a reciprocal blast approach. We generated gene lists for 19 signalling pathways, in two steps: (i) manual curation of main members of selected pathways, (ii) identification of their gene ID in the *S. purpuratus* genome via echinobase gene searches 137 and (iii) identification of *S. purpuratus* orthologs in *A. filiformis* using gene trees built with Generax [23] for each of our broccoli [24] gene family. Finally, the repertoire of luciferase-like genes was identified through reciprocal BLAST searches using the reference *Renilla* luciferase (GenBank: AAA29804) as initial query [5].

Hox and ParaHox genes

We first compiled a dataset of curated full length HOX protein sequences from *S. purpuratus* and HOX homeodomains from *B. floridae* and *S. kowaleskii* to search for homologous Hox genes in the brittle star. A comprehensive list of candidate Hox genes in brittle star was then constructed using two approaches: (i) a diamond blastp [25] of the curated Hox dataset against brittle star predicted proteins (ii) a miniprot [26] alignment of *S. purpuratus* HOX protein sequence against the brittle star genome sequence, to recover *Hox* genes potentially missed by the automatic annotation process. The same approach was used to identify *Hox* genes in *M. glacialis*. Finally, we built a molecular phylogenetic tree (**Extended Data Fig. 4**) with RAxML-NG [27] using the LG+G4+F model and 5 distinct starting parsimony trees, to reconstruct the evolutionary history of echinoderm Hox genes. We moreover validated correct assembly at the Hox cluster locus by inspection of nanopore reads coverage. We extracted ParaHox sequences and location in *A. japonica* from Ensembl Metazoa v56 [28], in *S. purpuratus* from Echinobase, *A. planci* from Ensembl Metazoa and similar approaches as for the Hox to identify ParaHox genes in *A. filiformis* and *M. glacialis*.

Supplementary Tables

Table S1: Accessions for *A. filiformis* RNA-seq datasets.
This table lists all RNA-seq datasets used and/or generated in this study, along with the corresponding SRA accession numbers. The datasets used for the genome annotation are also indicated.

Table S2: *A. filiformis* gene models properties.
This table presents the 30,267 predicted gene models, along with their properties, including: genomic location (chromosome or scaffold, start, end, strand columns), predicted gene name based on a blast with the swissprot database, predicted gene age, expression sample with maximal TPM (Max TPM, Max Sample) within the normalised "development and arm regeneration" dataset (**Table S1, Dataset S5**), cluster membership for the regeneration time course, differential expression in explant experiment, curated gene list membership, PFAM Domains, and a reduced set of associated Gene Ontology Terms.

Table S3: *A. filiformis* genes and signalling pathways.
Genes annotated to the 19 signalling pathways. The first 19 columns contain the 19 pathways, where a '1' in a given column represents membership to the pathway, additional columns contain the *A. filiformis* gene names and *S. purpuratus* orthologs.

Table S4: *A. filiformis* immune genes categories.
Breakdown of *A. filiformis* immune genes into receptors, signalling, transcription factors, effectors and homology-based predictions.

Table S5: List of DNA-binding PFAM domains.
PFAM domains used to identify putative TF-encoding genes.

Table S6: Enriched gene ontology terms in brittle star expanded and contracted gene families.
Gene ontology enrichment results for each brittle star arm regeneration co-expression cluster, with columns as follows: GO term ID, GO term description, GO dispensability score, enrichment, adjusted p-value (BH correction for multiple testing), corresponding gene families identifiers and brittle star genes.

Table S7: Gene expansion and contraction and expression in regeneration.
Co-expression regeneration cluster membership for regeneration and keratan sulfate genes, with columns as follows: co-expression cluster, fraction of gene family member in cluster, fraction of total genes in cluster, enrichment ratio and adjusted p-value (BH correction for multiple testing).

Table S8: Co-expression clusters and expression of known brittle star regeneration genes.

Cluster membership for previously investigated brittle star regeneration genes [29–31].

Table S9: Co-expression clusters and expression of novel brittle star regeneration genes.

List of core genes in each co-expression cluster as putative novel candidates.

Table S10: Enriched gene ontology terms in brittle star arm regeneration co-expression clusters.

Gene ontology enrichment results for each brittle star arm regeneration co-expression cluster, with columns as follows: GO term ID, GO term description, fraction of genes in the cluster annotated with the GO term, fraction of background genes annotated with the GO term, p-value, adjusted p-value (BH correction for multiple testing), q-value, corresponding genes and number of genes.

Table S11: Axolotl limb regeneration co-expression clusters.

Axolotl gene ID (AmexG_v6.0-DD assembly version) in each co-expression cluster.

Table S12: Enriched gene ontology terms in axolotl limb regeneration co-expression clusters.

Gene ontology enrichment results for each axolotl arm regeneration co-expression cluster, as in Table S10.

Table S13: Genes with conserved expression profiles during appendage regeneration in brittle star, axolotl and Parhyale.

List of genes co-expressed during regeneration in brittle star, axolotl and Parhyale, with columns as follows: species comparison in which the genes are conserved, co-expression clusters in these species, gene family ID, gene IDs and gene names, and curated gene list membership.

Table S14: Enriched gene ontology terms in genes with conserved expression profiles during appendage regeneration.

Gene ontology enrichment results as in Table S10.

Supplementary references

1. Parey, E. *et al.* Supplementary datasets for the brittle star A. filiformis genome. https://doi.org/10.5281/zenodo.10785182 (2024).
2. Mashanov, V. *et al.* Twinkle twinkle brittle star: the draft genome of Ophioderma brevispinum (Echinodermata: Ophiuroidea) as a resource for regeneration research. *BMC Genomics* **23**, 574 (2022).
3. Long, K. A., Nossa, C. W., Sewell, M. A., Putnam, N. H. & Ryan, J. F. Low coverage sequencing of three echinoderm genomes: the brittle star Ophionereis fasciata, the sea star Patiriella regularis, and the sea cucumber Australostichopus mollis. *Gigascience* **5**, 20 (2016).
4. Arshinoff, B. I. *et al.* Echinobase: leveraging an extant model organism database to build a knowledgebase supporting research on the genomics and biology of echinoderms. *Nucleic Acids Res.* **50**, D970–D979 (2022).
5. Delroisse, J. *et al.* A puzzling homology: a brittle star using a putative cnidarian-type luciferase for bioluminescence. *Open Biol.* **7**, (2017).
6. Marlétaz, F. *et al.* Analysis of the P. lividus sea urchin genome highlights contrasting trends of genomic and regulatory evolution in deuterostomes. *Cell Genom* **3**, 100295 (2023).
7. Oliveri, P., Davidson, E. H. & McClay, D. R. Activation of pmar1 controls specification of micromeres in the sea urchin embryo. *Dev. Biol.* **258**, 32–43 (2003).
8. Dylus, D. V. *et al.* Large-scale gene expression study in the ophiuroid Amphiura filiformis provides insights into evolution of gene regulatory networks. *Evodevo* **7**, 2 (2016).
9. Venturini, L., Caim, S., Kaithakottil, G. G., Mapleson, D. L. & Swarbreck, D. Leveraging multiple transcriptome assembly methods for improved gene structure annotation. *Gigascience* **7**, (2018).
10. Grabherr, M. G. *et al.* Full-length transcriptome assembly from RNA-Seq data without a reference genome. *Nat. Biotechnol.* **29**, 644–652 (2011).
11. Wu, T. D. & Watanabe, C. K. GMAP: a genomic mapping and alignment program for mRNA and EST sequences. *Bioinformatics* **21**, 1859–1875 (2005).
12. Dobin, A. *et al.* STAR: ultrafast universal RNA-seq aligner. *Bioinformatics* **29**, 15–21 (2013).
13. Pertea, M. *et al.* StringTie enables improved reconstruction of a transcriptome from RNA-seq reads. *Nat. Biotechnol.* **33**, 290–295 (2015).

14. Niknafs, Y. S., Pandian, B., Iyer, H. K., Chinnaiyan, A. M. & Iyer, M. K. TACO produces robust multisample transcriptome assemblies from RNA-seq. *Nat. Methods* **14**, 68–70 (2017).
15. Boutet, E., Lieberherr, D., Tognolli, M., Schneider, M. & Bairoch, A. UniProtKB/Swiss-Prot. *Methods Mol. Biol.* **406**, 89–112 (2007).
16. Stanke, M. & Waack, S. Gene prediction with a hidden Markov model and a new intron submodel. *Bioinformatics* **19 Suppl 2**, ii215–25 (2003).
17. Levy Karin, E., Mirdita, M. & Söding, J. MetaEuk-sensitive, high-throughput gene discovery, and annotation for large-scale eukaryotic metagenomics. *Microbiome* **8**, 48 (2020).
18. Mapleson, D., Venturini, L., Kaithakottil, G. & Swarbreck, D. Efficient and accurate detection of splice junctions from RNA-seq with Portcullis. *Gigascience* **7**, (2018).
19. Haas, B. J. *et al.* Improving the Arabidopsis genome annotation using maximal transcript alignment assemblies. *Nucleic Acids Res.* **31**, 5654–5666 (2003).
20. Sea Urchin Genome Sequencing Consortium *et al.* The genome of the sea urchin Strongylocentrotus purpuratus. *Science* **314**, 941–952 (2006).
21. Alié, A. *et al.* The ancestral gene repertoire of animal stem cells. *Proc. Natl. Acad. Sci. U. S. A.* **112**, E7093–100 (2015).
22. McGinnis, S. & Madden, T. L. BLAST: at the core of a powerful and diverse set of sequence analysis tools. *Nucleic Acids Res.* **32**, W20–5 (2004).
23. Morel, B., Kozlov, A. M., Stamatakis, A. & Szöllősi, G. J. GeneRax: A Tool for Species-Tree-Aware Maximum Likelihood-Based Gene Family Tree Inference under Gene Duplication, Transfer, and Loss. *Mol. Biol. Evol.* **37**, 2763–2774 (2020).
24. Derelle, R., Philippe, H. & Colbourne, J. K. Broccoli: Combining Phylogenetic and Network Analyses for Orthology Assignment. *Mol. Biol. Evol.* **37**, 3389–3396 (2020).
25. Buchfink, B., Reuter, K. & Drost, H.-G. Sensitive protein alignments at tree-of-life scale using DIAMOND. *Nat. Methods* **18**, 366–368 (2021).
26. Li, H. Protein-to-genome alignment with miniprot. *Bioinformatics* **39**, btad014 (2023).
27. Kozlov, A. M., Darriba, D., Flouri, T., Morel, B. & Stamatakis, A. RAxML-NG: a fast, scalable and user-friendly tool for maximum likelihood phylogenetic inference. *Bioinformatics* **35**, 4453–4455 (2019).

28. Yates, A. D. *et al.* Ensembl Genomes 2022: an expanding genome resource for non-vertebrates. *Nucleic Acids Res.* **50**, D996–D1003 (2022).

29. Czarkwiani, A., Taylor, J. & Oliveri, P. Neurogenesis during Brittle Star Arm Regeneration Is Characterised by a Conserved Set of Key Developmental Genes. *Biology* **11**, (2022).

30. Piovani, L., Czarkwiani, A., Ferrario, C., Sugni, M. & Oliveri, P. Ultrastructural and molecular analysis of the origin and differentiation of cells mediating brittle star skeletal regeneration. *BMC Biol.* **19**, 9 (2021).

31. Czarkwiani, A., Dylus, D. V., Carballo, L. & Oliveri, P. FGF signalling plays similar roles in development and regeneration of the skeleton in the brittle star Amphiura filiformis. *Development* **148**, (2021).
